# Agricultural commercialization and nutrition revisited: Empirical evidence from three African countries

**DOI:** 10.1016/j.foodpol.2016.09.020

**Published:** 2017-02

**Authors:** Calogero Carletto, Paul Corral, Anita Guelfi

**Affiliations:** a Development Data Group, World Bank, United States; b Poverty and Equity Global Practice, World Bank, United States; c University of Rome ‘Tor Vergata’, Italy

**Keywords:** Agricultural commercialization, Cash crops, Nutrition, Africa

## Abstract

The transition from subsistence to commercial agriculture is key for economic growth. But what are the consequences for nutritional outcomes? The evidence to date has been scant and inconclusive. This study contributes to the debate by revisiting two prevailing wisdoms: (a) market participation by African smallholders remains low; and (b) the impact of commercialization on nutritional outcomes is generally positive. Using nationally representative data from three African countries, the analysis reveals high levels of commercialization by even the poorest and smallest landholders, with rates of market participation as high as 90%. Female farmers participate less, but tend to sell larger shares of their production, conditional on participation. Second, we find little evidence of a positive relationship between commercialization and nutritional status. As countries and international agencies prioritize the importance of nutrition-sensitive agriculture, better understanding of the transmission channels between crop choices and nutritional outcomes should remain a research priority.

## 1 Introduction

According to conventional wisdom, the transition from subsistence (or semi-subsistence) to commercial agriculture represents a key ingredient for the economic development of low-income countries. By exploiting comparative advantages, agricultural commercialization enhances trade and efficiency, leading to economic growth and welfare improvement at the national level. This is further expected to initiate a virtuous cycle which raises household income, thus improving consumption, food security and nutritional outcomes inside rural households.

Yet, this mainstream, beneficial view of agricultural commercialization has also been challenged several times since the 1970s, with a large body of literature in the 1970s and the first half of the 1980s^1^ emphasizing the adverse effects on households’ welfare and nutrition, especially on the poorest groups of the rural population and the most vulnerable individuals within the household who are often considered unable to reap the benefits of increased market orientation.^2^ The concerns related especially to their food security and nutritional outcomes.^3^ While many of these studies displayed a pronounced degree of ideology,^4^ they also highlighted the need to better understand the underlying linkages between crop production, commercialization, income, consumption and nutrition at the household level.

Against this background, the International Food Policy Research Institute (IFPRI) revisited the issue,^5^ using a more scientific and systematic approach which consisted of three components: (i) the development of a conceptual framework articulating the linkages between commercialization and nutrition; (ii) a better research design to compare commercialized and non-commercialized households; and (iii) the use of a cross-country comparative approach based on six different but comparable country micro-level analyses^6^ carried out using a common research design. The IFPRI studies also mitigated the traditional assumption of a dichotomy – and hence a necessary competition – between cash and staple crops, which had deeply influenced the way agricultural commercialization had been conceived and measured in most of the previous literature.^7^

Unlike many of the previous studies, the majority of IFPRI country studies found generally a positive, though small, impact of agricultural commercialization on the nutritional status of rural households, where the positive relationship was assumed to operate primarily through the linkages between household income, household caloric intake, and child caloric intake. Nevertheless, as the authors of the studies acknowledged, several limitations remained: “Econometrically, a common practice is to estimate a set of reduced form equations with an extended list of exogenous explanatory variables that affect any of the structural relations. This approach is not followed in this book, in part because of data limitations (von Braun and Kennedy, 1994; Ch. 2, p. 24).”

Since then, there has been little new empirical evidence^8^ on the links between agricultural commercialization and nutrition,^9^ despite the implementation of numerous expensive projects to promote market-oriented crops, based on the assumption of a beneficial nutritional effect.

In the spirit of the other papers in this volume, this study revisits two prevailing wisdoms. First, participation in market activities by smallholders is low. Second, the impact of commercialization on nutritional outcomes is generally positive. In doing so, the paper reconsiders the quantification and characterization of agricultural commercialization and provides new, systematic evidence on its relationship with nutritional outcomes in three Sub-Saharan countries. In particular, it uses recent panel surveys from Malawi, Tanzania and Uganda conducted under the Living Standards Measurement Study-Integrated Surveys on Agriculture (LSMS-ISA) program. Unlike in most previous studies, these surveys are nationally representative, which enables a more systematic comparison across different settings and also allows one to better control for a number of the endogeneity issues that arise in estimating the impact of commercialization on nutritional outcomes. The study further aims to capture the heterogeneity implicit in the commercialization choices of different smallholder households. For example, Duflo and Udry (2004) suggest that income from different crops as well as income from different plot owners may serve distinct purposes within the household and thus have different impacts. Using individual-level crop data, we are able to differentiate the impact of commercialization based on the gender of the farmers and the type of crop mix grown and sold, which are both assumed to affect the relationship between commercialization and nutritional outcomes.

The paper is organized as follows. Sections 2, 3 provide a brief overview of the literature and a short description of the data, respectively. Section 4 profiles commercialization in the three countries by constructing an index of commercialization at the household and crop levels. Section 5 descriptively explores the relationship between agricultural commercialization and nutritional outcomes. Then the section presents an econometric strategy and the main findings. Finally, conclusions are presented in Section 6.

## 2 Agricultural commercialization and nutrition: a brief literature review

The empirical literature on the nutritional outcomes of agricultural commercialization can be grouped into three strands: (i) a wide and heterogeneous set of research projects carried out before the launch of the IFPRI agenda; (ii) the IFPRI work between 1986 and 1994; (iii) a few studies devoted to the topic starting from the early 1990s.

A review of the first wave of studies fails to settle the debate on the linkages between agricultural commercialization and nutrition. As shown in Table 1 (which reports the literature review carried out in von Braun and Kennedy, 1986^10^), results are confusing and ambiguous, with the same crop having opposite effects both between and within countries. Studies in this period usually lacked a proper conceptual framework, adopting instead a “black-box” approach which did not articulate the underlying channels leading to various outcomes. The main approach was a comparison of nutritional outcomes between cash crop adopters and non-adopters. The evidence was often anecdotal and based on country case studies, making it impossible to compare results both across and within countries. In most studies, the definition and measurement of commercialization was subjective (based on the adoption or non-adoption of a given list of cash crops).

Subsequently, the IFPRI studies also developed a conceptual framework to articulate the complex set of linkages between the process of agricultural commercialization and the nutritional and health status at the household level. In particular, they examined how agricultural commercialization affected each of the four key steps between national food production and individual nutritional outcomes, identified by Pinstrup-Andersen in the early 1980s,^11^ i.e. “national/community food availability”, the “ability and desire of households to obtain food”, “intrahousehold food distribution” and “health and sanitary factors”.

First, the decision to adopt a market-oriented production system is expected to influence the degree of food availability at the national, community and household levels. Factors such as competition among limited resources (such as land, labor and capital), the amount of food imports and aid, the degree of diversity of available foods and the presence of seasonal and irregular fluctuations may be influenced by a rise in market orientation in smallholder farmers. Through this channel they may impact national or regional food availability, which, by affecting food prices, may have relevant nutritional implications. However, national food sufficiency can be a poor indicator of household nutritional status, as “food may be plentiful but the poor may still be unable to access it”.^12^ Thus, at the household level, it is important to look at the ability of each household to effectively obtain food.^13^ This ability varies depending on the effects of the commercialization process on several factors, among which the most important one is household income.^14^

If real income increases at the household level, it could then stimulate a virtuous cycle through which smallholder farmers can enhance their level of food consumption. While necessary, the rise in real income, is again not sufficient to improve household consumption. Indeed, households must have the “desire to obtain available (and nutritious) food”, a condition which is often not satisfied due to intra-household factors, as individual household members are likely to have different income elasticities.^15^ Furthermore, even if additional income is spent on food, intra-household food consumption could be heterogeneously distributed among family members, with children and women often relatively penalized compared with adult males. Furthermore, a high marginal propensity to spend on food does not automatically imply a high marginal propensity to consume calories. Households may choose to “diversify into a more varied, higher cost diet rather than simply using the income to increase energy intake”.^16^ Finally, a crucial role is played by the potential impact of changes in income on health and sanitary factors, as increased income from commercialization may be invested towards improved water sources and/or sanitation both at the household and community level.

Two key results emerged from the IFPRI studies^17^:

(i). The impact of agricultural commercialization on the nutritional status^18^ of rural households was found to be mostly positive, though rather small in magnitude. This positive relationship mainly operated through the linkages between household income, household caloric intake and child caloric intake. Cash crop adoption generally increased real incomes, thereby stimulating a virtuous cycle whereby higher incomes were used to increase food consumption, which benefited on average both the household in general and the children in particular.
(ii). The effect of agricultural commercialization on nutrition will depend on a number of conditioning, complementary factors both at the macro and micro level, making the adoption of commercial crops more or less remunerative and sustainable. The complex set of linkages characterizing the commercialization process and its impact on household welfare and nutrition suggest that several different scenarios can emerge depending on the factors dominating in each circumstance. According to the study, a key role must be played by policies (both macro and micro^19^) aiming at enhancing beneficial outcomes while minimizing adverse ones.^20^

Two decades after the publication of von Braun and Kennedy (1994), the somewhat positive view on the impact of commercialization of agriculture on welfare outcomes is still prevalent. In fact, since then, there have been only few new studies explicitly looking at the link between agricultural commercialization and nutrition and the evidence remains inconclusive.^21^ The present study is an attempt to shed some light on this rather controversial, yet important, relationship using data from three countries in sub-Saharan Africa.

## 3 Data description

To revisit the link between agricultural commercialization and nutrition, we use the data from the nationally representative household panel surveys collected in three countries (Malawi, Tanzania, Uganda) under the Living Standards Measurement Study – Integrated Surveys in Agriculture (LSMS-ISA) initiative.^22^

All households were administered a multi-topic household questionnaire, and those involved in any agricultural activities were also administered a detailed agricultural module. The surveys collect detailed crop and plot level information, as well as a rich set of socioeconomic characteristics and information on child anthropometrics. Given agricultural production estimates at the plot level as well as the identification of the plot manager, the level of commercialization can also be computed at the individual level.

The surveys was conducted throughout the year, though each household was only interviewed once. To adjust for this difference in the timing of the interview, when calculating the commercialization index reported sales were annualized using imputation methods (see Appendix A for details).^23^

Given our focus, our sample includes only farming households, defined as households who reported involvement in agricultural activities through ownership and/or cultivation of land in the most recently completed agricultural season.^24^ The descriptive analysis presented in this paper in Sections 3, 4, 5 are based on the full samples of the baseline surveys which were carried out in 2010/11 in all three countries. Our final sample at baseline thus consists of 9894 households in Malawi, 2074 households in Tanzania and 1788 households in Uganda. The panel component is introduced in Section 5.1 in order to address some of the econometric challenges of the estimation. After excluding the non-panel and non-farming households, the final sample size of the panel used for the estimations in each country is 2222, 1744 and 1587 farming households in Malawi, Tanzania and Uganda, respectively.^25^ Overall, sample attrition between the two waves was rather low.

As shown in Table 2, the great majority of households in our sample are male-headed, with the share of the female-headed households ranging from 25% (Malawi and Tanzania) to 30% (Uganda). Significant differences emerge in terms of educational attainment: in Malawi about 78.8% of the rural households did not receive any type of formal education, while this percentage amounts to about 30.2 and 18.8% in Tanzania and Uganda, respectively. In the latter two countries, the large majority of rural households attained at least a primary level of education.

Another source of significant variation between the three countries is the size of land available to farmers. Average land size is slightly below 1 ha in Malawi, compared with 2.3 and 2.6 ha respectively in Uganda and Tanzania. The three countries also look different in terms of crop diversification, with Malawian and Tanzanian families choosing to plant 2 types of crops on average, compared with around 4 in Uganda.

Table 2 also details the differences between selling households and non-selling households. We find that close to 90% of households in Malawi engage in sales, compared to 80% in Uganda and 68% in Tanzania. Not surprisingly, when broken down by land size quintiles, the incidence of households selling any crop is monotonically increasing, with larger farmers selling greater shares. Overall, these preliminary figures suggest that in all three countries, the majority of farm households sell part of their production for both staple and cash crops. In this respect, our data suggests that the vast majority of commercialized households are only producing (and selling) food crops, from a minimum of 79% in Uganda to a maximum of 91% in Tanzania. Most remaining commercializers are growing and marketing both food and cash crops (9% in Tanzania, 21% in Uganda and 16% in Malawi), whereas those focusing only on the production and sale of non-food crops represent less than 1% in each country.

## 4 Measuring agricultural commercialization in sub-Saharan Africa

In the previous section, we demonstrated the high incidence of participation in market activities among even the smallest of smallholders. However, how much do those farming households sell? Who are the individuals in the household most involved in selling and what products do they sell the most? How best to define and measure the actual degree of agricultural commercialization in a given country has been much debated in recent decades. In this paper we use the Household Crop Commercialization Index (**CCI**), introduced by Strasberg et al. (1999) and Govereth et al. (1999), which is defined as:

(1) $${\mathrm{CCI}}_{i}=[\mathrm{Gross}\;\mathrm{value}\;\mathrm{of}\;\mathrm{crop}\;{\mathrm{sales}}_{\mathrm{hhi}\text{,}\mathrm{year}\;j}/\mathrm{Gross}\;\mathrm{value}\;\mathrm{of}\;\mathrm{all}\;\mathrm{crop}\;{\mathrm{production}}_{\mathrm{hhi}\text{,}\mathrm{year}\;j}]*100$$

Though not without its own shortcomings,^26^ a measure on the output side is able to capture a “household’s ‘revealed’ marketing behavior,^27^” and can be seen as relatively easier to collect,^28^ while lending “itself well to an empirical test within a regression framework^29^”.

According to this measure, the process of agricultural commercialization can be represented by a continuum ranging from pure subsistence (**CCI_i_** = **0**) to a completely commercialized production system (**CCI_i_** = **100**). Its main advantage is that it permits to go beyond the traditional dichotomies of sellers versus non-sellers, or between staple and cash crop producers. In fact, it adds an additional dimension to the discussion, i.e. how much of their harvest households choose to sell - while still being relatively easy to compute.

For the countries studied here, the CCI amounts to 17.6% in Malawi, 26.3% in Uganda and 27.5% in Tanzania on average (Table 3). When restricting the sample to farm households reporting any sales (conditional CCI), it rises slightly to 19.6, 40.4 and 33% respectively. Also, the degree of commercialization increases with farm size, likely reflecting larger surpluses of edible crops and/or greater adoption of cash crops by farmers with larger landholdings.

Relying on individual-level data, we are also able to compute CCI measures separately for male and female farmers.^30^ At first glance, female farmers appear to commercialize considerably less of their harvest than their male counterparts. However, when focusing only on those individuals who report selling (conditional CCI), the difference disappears or even reverses. In fact, among sellers, females appear to be more commercially oriented than their male counterparts both in Malawi (selling 31% of their production vs. 22 among male farmers) and in Uganda (37 vs. 35). Meanwhile, in Tanzania both genders have virtually the same CCI, at 43%.

Breaking down our unconditional gender-disaggregated CCI measure by farm size^31^ confirms a positive relationship between commercialization and landholdings for both male and female farmers. However, the gender gap in commercialization appears to increase, particularly in Malawi where among farmers with more than 1 ha, male unconditional CCI is almost double that of their female counterparts. This gender gap across farm sizes is not as stark in the other two countries. The larger gender gaps in Malawi may reflect greater restrictions for female farmers on fully participating in the production and sale of tobacco (the main cash crop in Malawi), as well as constraints to accessing more land resources to allow for greater crop diversification.

To assess the degree of commercialization of these staple and other food crops, we thus proceed to construct separate CCIs to reflect the degree of commercialization of food items versus non-edible items. As non-edible items are planted in most instances with the primary purpose of selling, it is not surprising to find the CCI for households who plant these crops to be as high as 91% for tobacco in Malawi^32^ or 87% for coffee in Uganda.

Farm households, however, do not only sell traditional cash crops, i.e. crops grown almost exclusively for sale. Table 4 shows that households in all three countries to a large extent are also involved in the sale of traditional staple crops such as maize and/or cassava. However commercialization of most food crops remains low although, with the exception of Malawi, households who do choose to sell, sell a considerable portion of their harvest. In Malawi, on the contrary, food crops like maize and cassava are sold by many households, but it is only done in small quantities. This relatively high incidence of small quantities of maize sales is the reason why the country’s CCI is low.

In the countries studied, there is commercialization of staple items such as maize (5% in Malawi, 16 in Tanzania and 20 in Uganda) and beans (10% in Malawi, 20 in Tanzania and 13 in Uganda). Overall, the share of food crops sold is 10% in Malawi, 24% in Tanzania and 23% in Uganda. Looking at the shares of food crop sold by farm size, as expected, farmers with larger landholdings tend to sell larger shares of their food production, reflecting greater surpluses, although in countries like Malawi, the share remains rather low, at 14% even for farmers with more than 2 ha of land.

**Graph 1:**
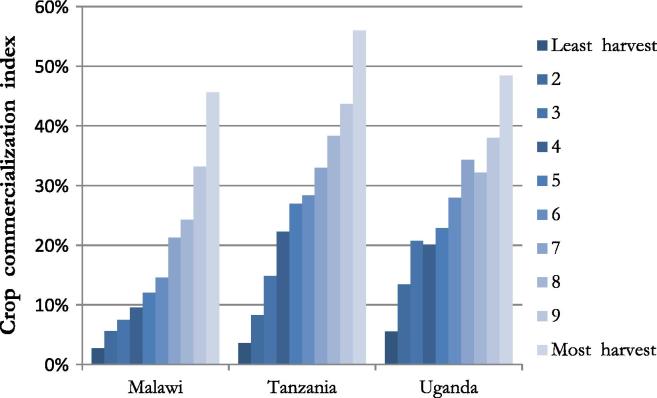
Avg. agricultural commercialization by harvest value deciles.

Finally, as expected, those with greater harvests (measured in monetary value) tend to have higher levels of commercialization in all three countries. Graph 1, presents the average CCI by harvest value deciles, and illustrates the level of commercialization across the harvest value distribution. Even households with the lowest harvest values engage the market.

## 5 Exploring the relationship between agricultural commercialization and nutrition

In this section, we investigate the nexus between the degree of agricultural commercialization and the nutritional status of farm households. Three indicators are used to measure household nutritional status: (i) children’s anthropometric measures (measured both in terms of percentage of children stunted, wasted and underweight, and through the computations of Z-scores), (ii) household per capita food expenditure, and (iii) household per capita caloric consumption.

**Graph 2:**
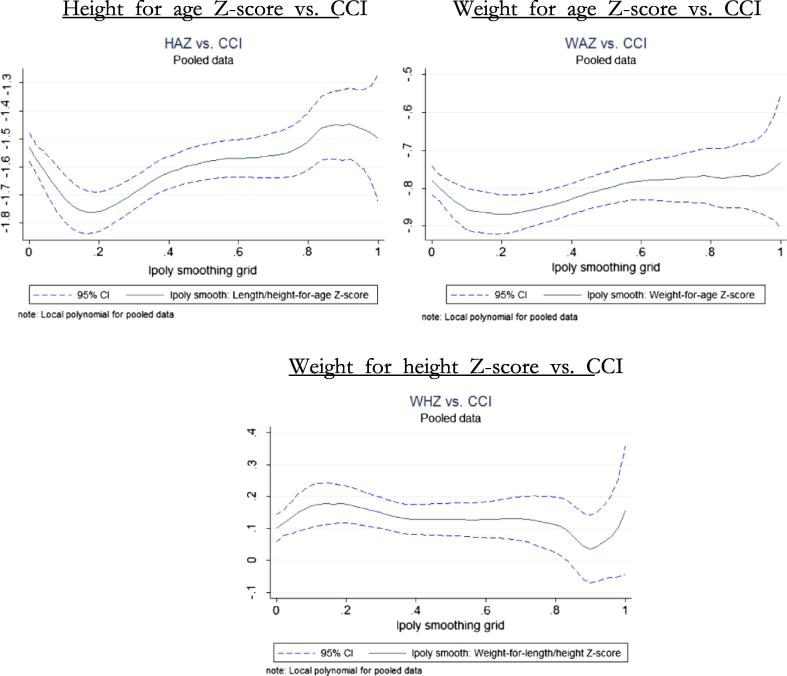
Agricultural commercialization and nutrition: pooled sample.

*Source:* Own computations on LSMS -ISA.

Table 5 suggests high levels of malnutrition in all countries, with an incidence of stunting among children under five years old of about 42% in Tanzania, compared to 36% in Uganda and 31% in Malawi. Similarly, the share of children wasted amounts to 6.2, 3.2 and 3.6% in Tanzania, Uganda and Malawi respectively. In terms of average caloric consumption, Tanzania exhibits average per capita caloric consumption of 2044 kilocalories, compared with 2536 in Malawi and 2243 in Uganda. Across countries, there is no clear relationship between the nutritional outcomes and the degree of commercialization (as proxied by the CCI quintiles) and the different nutritional indicators, with the exception of stunting in Tanzania. Similarly, no clear trends emerge when the degree of agricultural commercialization is correlated with children’s anthropometrics as measured through Z-scores (see Graph 2).

The absence of a correlation with child anthropometry might be partially attributable to the smaller sample size of children, particularly for Tanzania and Uganda. Pooling the three country samples and running a local polynomial non-parametric regression (without any control variables) a slightly upward gradient with commercialization emerges for the height-for age (stunting) and weight-for-height (wasting) measures, suggesting a some positive correlation between child nutritional outcomes and household’s commercialization. In the remainder of the paper, we explore these relationships in more detail using a multivariate framework based on the pooled sample.

### 5.1 Empirical strategy and main results

In order to analyze further how commercialization may affect nutritional outcomes, more directly through changes in food consumption and more indirectly through changes in income, we estimate a set of models first at the individual level to investigate how CCI impacts child nutritional status and then at the household level with the aim of exploring how CCI correlates to household per capita expenditure.

Specifically, in Table 6 we report selected findings of the estimated impact of commercialization on child anthropometrics. In this instance the sample has been pooled due to the reasons mentioned in the previous section. Table 7 illustrates estimates of the probability of a child being stunted, wasted, or underweight. For each model, we use five different specifications based on different characterizations of household commercialization and thus how we introduce the CCI. The aim is to determine if increased sales of the household’s harvest could be related to observed anthropometric outcomes. The owner of the revenue from the sales could be of importance^33^; specifications that take this into account are also included. In the first specification of each model (Column 1) we include the overall household commercialization index. In column 2, we add to the previous specification the share of the household CCI accruing to female farmers within the household. In column 3, we introduce yet another specification of the gender CCI by using the Female CCI. In a similar fashion, to account for the potentially differential impact of commercialization of food commodities, in column 4 and 5 we introduce two variants of the Food CCI, first by adding to the total CCI the share of total CCI deriving from the sale of food crops and then by replacing the total CCI with the Food CCI.

In each model and specification, the common correlates are: gender of head, age of head, education of the head, natural logarithm of land holdings, natural logarithm of land holdings squared, the natural logarithm of the household’s harvest value, annual average rainfall in millimeters, and the child’s age in months as well as the child’s gender.

The key variables in this model (CCI and its variants) are in all likelihood endogenous due to simultaneous causality between the dependent variables and commercialization. Additionally, it is possible that several common unobservable factors impact both kinds of outcomes. In order to address these potential endogeneity issues, the panel component of the data is used by estimating fixed effects models. Naturally, time-varying covariates which are not controlled for by the fixed-effect model still present a potential problem.

### 5.2 Anthropometrics with pooled sample of children

In order to analyze how commercialization relates to children’s nutritional outcomes, we focus on the pooled sample of children present in both waves, who were older than 6 months during the first round and younger than 60 months old by the second wave. We run both an individual fixed effects linear model on Z-scores as well as a random effects logit model on the probability of being stunted, wasted or underweight.

The fixed effects results in Table 6 are quite consistent for total CCI and its variants, with the coefficients being largely not significant. More explicitly, there is no relationship between anthropometric outcomes and CCI, both as a total and when disaggregated by food and non-food products. The few exceptions are in the WAZ and WHZ models where, at equal levels of total household commercialization, the share of CCI accruing to women is negative and significant. This suggests that greater involvement by women may result in some negative effect for short-term nutritional outcomes. However, in light of the rather small sample size of children in the panel, these results should be taken with some caution. Finally, the level of per capita expenditure in the household is also not significantly related to Z-scores.

The probability of a child being stunted, wasted, or underweight is modelled in Table 7. In line with the results presented in Table 6, the coefficients only show a significant and negative effect of greater commercialization by women on short-term nutritional indicators, likely a reflection of the potentially deleterious effect of lower levels of child care on child nutritional status. Per capita expenditure in this instance does seem to play a role, with an increase in expenditure negatively related to the child’s likelihood of being stunted, and underweight. The relationship, however, is not significant for wasting.

In Table8 we analyze the relationship between commercialization and per capita food expenditures. With the above mentioned caveats on the potential endogeneity of some of the regressors,^34^ overall we fail to find any clear pattern, and the findings seem to diverge only slightly across countries. For instance, looking at the first columns, we find little evidence of a relationship between CCI and food expenditures, except for Uganda where the coefficient is negative and marginally significant. Also, in Uganda, the negative coefficient on female CCI is still marginally significant though somewhat larger than for the total CCI. All other coefficients provide little support to the existence of a relationship between commercialization, in its different specifications, and food expenditures in any of the countries analyzed.

In Table 9 we report the same coefficients by regressing household total per capita expenditure on the same set of regressors and various CCI specifications. The results we found for food expenditures for Malawi carry over to this specification with little evidence of any impact of commercialization on total expenditures. This lack of impact may be due to the fact that while commercialization is widespread across farmers, sales often involve small amounts which fail to have a significant impact on total household per capita expenditures.^35^

## 6 Main conclusions

Despite the inconclusiveness of the available empirical evidence to date, agricultural commercialization among poor smallholders continues to be heralded as an effective solution to reduce poverty, improve household food and nutrition security, and foster growth in rural areas. Based on new comparable data from across sub-Saharan Africa which enables the calculation of commercialization indexes at the individual and crop level, this paper contributes to the ongoing debate by investigating the relationship between increased agricultural commercialization and several nutritional indicators in three African countries, differentiated by gender and types of crops sold.

Against conventional wisdom, the data reveal very high levels of commercialization by even the poorest and smallest land holders, with rates of market participation as high as 90% in Malawi. Similarly, against common perceptions, a considerable portion of this market presence is driven by the sale of staple and other food crops and not necessarily by traditional cash crops. This is in part due to the fact that the great majority of smallholders are still specializing in the production of food crops (between 80 and 90% in the three countries analyzed), with only a relatively small share cultivating both food and traditional cash crops. However, in most cases, particularly in Malawi, market participation only involves the sale of relatively small quantities of own food production, resulting in low food CCI-- 10% for the entire sample and only 14% among the largest farmers.

Another important finding of the cross-country analysis is that although female farmers appear to participate less in market activities, when they do participate, they tend to sell larger shares of the production under their control relative to their male counterparts.

In line with previous research, we also find little evidence of a relationship between increased commercialization and improved nutritional status. The only exception is a weak negative relationship between the portion of commercialization accruing to females and short-term nutritional indicators, which could be the results of the negative effect of greater female market participation on time allocated to child care and homemaking.

Nonetheless, these findings should still be taken with caution, as we are still unable to fully control for the potential simultaneity of the CCI and total harvest value and other variables, despite the use of panel data. That said, the arguably more robust and representative evidence presented here is in line with the bulk of evidence to date, and yet another piece of empirical evidence of the weak association between increased commercialization and improved food security and nutritional outcomes.

## Figures and Tables

**Table A1:** Sample composition.

		Malawi	Tanzania	Uganda
Maize	Observations	9578	1510	1172
	Obs. Selling	1520	489	441

Legumes	Observations	4224	1172	256
	Obs. Selling	1279	488	

Tubers	Observations	479	360	1832
	Obs. Selling	200	101	

Grains	Observations	1104	541	764
	Obs. Selling	126	214	241

Rice	Observations	619	351	N/A
	Obs. Selling	378	221	

Ground nut	Observations	2466	N/A	494
	Obs. Selling	855		193

Cassava	Observations	1452	N/A	N/A
	Obs. Selling	257		

Banana	Observations	N/A	N/A	1202
	Obs. Selling			533

Beans	Observations	N/A	N/A	1208
	Obs. Selling			399

**Table 1:** Summary of micro-studies on income and nutritional effects of cash crop production reviewed in von Braun and Kennedy (1986).

Study	Country	Crop	Effects on	
			Family consumption	Nutritional Status
Lev (1981)	Tanzania	Coffee, Bananas	Positive	n.a.
Hitchings (1982)	Kenya	Tea, Coffee, Cotton, Pyrethrum, Sugarcane	n.a.	Positive, Positive, Neutral, Neutral, Negative
Rabeneck (1982)	Kenya	Coffee, Staples	n.a.	Positive
Fleuret and Fleuret (1983)	Kenya	Coffee, Vegetables	n.a.	Positive
Gross and Underwood (1971)	Brazil	Sisal	Negative	n.a.
Dewey (1981)	Mexico	Cocoa, Sugarcane	n.a.	Negative
Hernandez et al. (1974)	Mexico	Cocoa, Sugarcane	n.a.	Negative
Lambert (1978)	Papua New Guinea	Coffee	Negative	n.a.
Harvey and Heywood (1983)	Papua New Guinea	Coffee	Positive	Positive

**Table 2:** Main sample characteristics.

Sample characteristics	Malawi			Tanzania			Uganda		
	All	Sellers	Non sellers	All	Sellers	Non sellers	All	Sellers	Non sellers
**# of households**	9894	8727	1167	2074	1335	739	1788	1415	373

*of which:*									
Male headed (%)	75.4	75.7	74.7	73.7	72.7	71.7	70.7	69.7	68.7
Female headed (%)	24.6	24.3	25.3	26.3	27.3	28.3	29.3	30.3	31.3

**Education (%)**									
– None	78.8	78.2	83.8	30.2	28.7	33.3	18.8	16.6	27.7
– Primary	9.1	9.4	6.9	62.9	65.4	57.5	58.2	59.0	55.2
– Secondary	11.0	11.2	8.7	6.8	5.8	9.1	18.2	19.4	13.7
– Tertiary	1.1	1.2	0.6	0.1	0.1	0.1	4.8	5.1	3.4

HH head age	43.1	43.7	48.6	47.3	51.4	47.0	46.7	48.1	
	(16.51)	(16.42)	(17.28)	(15.76)	(15.25)	(16.45)	(15.73)	(15.51)	(16.54)
HH size	4.7	4.7	4.5	5.5	5.6	5.5	5.3	5.4	5.1
	(2.17)	(2.18)	(2.08)	(2.93)	(2.93)	(2.94)	(2.62)	(2.62)	(2.63)
CDR	0.8	0.8	0.8	0.7	0.7	0.7	0.8	0.8	0.8
	(0.70)	(0.70)	(0.73)	(0.68)	(0.69)	(0.67)	(0.76)	(0.75)	(0.76)
Distance market (Kms)	7.9	7.9	8.0	75.5	78.7	68.8	31.6	31.4	32.4
	(5.31)	(5.32)	(5.25)	(50.97)	(53.09)	(45.46)	(17.79)	(16.87)	(21.02)
Distance pop. Center (Kms)	36.0	35.8	38.2	51.2	54.0	45.2	25.2	24.1	29.8
	(20.06)	(20.03)	(20.19)	(39.00)	(38.99)	(38.36)	(16.83)	(15.02)	(22.01)
P.c. food expenditure (USD)	0.52	0.53	0.42	0.63	0.63	0.63	0.40	0.42	0.35
	(0.34)	(0.35)	(0.29)	(0.35)	(0.34)	(0.36)	(0.26)	(0.27)	(0.25)
P.c. kcal Consumption	2536	2554	2383	2044	2078	1972	2243	2317	1954
	(2305.56)	(2239.66)	(2807.45)	(867.95)	(858.17)	(884.73)	(1567.90)	(1573.86)	(1512.01)
Hired labor (days)	4.4	4.8	1.5	8.5	10.8	3.4	16.2	17.9	9.7
	(17.27)	(18.08)	(6.47)	(25.32)	(29.57)	(10.42)	(35.31)	(37.20)	(25.63)
Land owned (Ha)	0.9	1.0	0.6	2.6	3.0	1.7	2.3	2.5	1.5
	(13.13)	(13.87)	(0.61)	(4.59)	(5.27)	(2.34)	(12.76)	(14.15)	(3.83)
# crops harvested	2.2	2.3	1.5	2.1	2.3	1.6	3.8	4.2	2.4
	(1.12)	(1.09)	(1.11)	(1.12)	(1.14)	(0.85)	(1.87)	(1.80)	(1.45)
# crops sold	1.7	1.8	0.0	1.0	1.5	0.0	1.8	2.3	0.0
	(1.02)	(0.90)	0.00	(0.94)	(0.76)	0.00	(1.49)	(1.31)	0.00
HH Harvest value (USD)	269.92	292.28	76.91	244.65	314.20	96.63	215.03	254.46	60.06
	(731.34)	(768.50)	(110.90)	(500.71)	(429.69)	(599.61)	(334.09)	(361.59)	(79.97)
HH revenue (USD)	102.28	114.13	0.00	112.23	164.96	0.00	85.59	107.38	0.00
	(542.64)	(572.03)	0.00	(286.60)	(334.75)	0.00	(217.84)	(239.16)	0.00
AG Income (USD)	285.18	308.40	84.72	281.35	350.66	133.84	234.07	272.07	84.77
	(738.08)	(775.05)	(130.67)	(534.48)	(472.76)	(621.66)	(354.33)	(378.56)	(167.05)
HH Days worked	125.30	129.00	93.36	149.32	161.51	123.38	134.10	143.74	96.22
	(116.28)	(119.63)	(74.64)	(146.35)	(150.35)	(133.91)	(106.10)	(109.05)	(83.57)

**Table 3:** CCI by chosen characteristics.

	CCI		
	Malawi	Tanzania	Uganda
Country average	17.6	27.5	26.3
Country average (conditional on sales)	19.6	40.4	33.0
Female headed	10.8	20.3	20.7
Female headed (conditional on sales)	12.2	33.7	28.7
Male headed	19.8	29.8	28.6
Male headed (conditional on sales)	22.0	42.3	34.5
Female farmers	9.0	19.1	23.0
Female farmers (conditional on sales)	30.6	42.9	37.0
Male farmers	19.8	30.8	27.0
Male farmers (conditional on sales)	21.7	42.8	34.6

*By land size*			
– Less than 0.5 ha	9.9	15.4	20.8
– Between 0.5 and 1 ha	19.8	21.6	25.3
– Between 1 and 2 ha	28.8	26.2	28.5
– 2 ha or more	34.8	34.8	30.7

**Table 4:** The degree of HHs’ agricultural commercialization by type of crop.

Crop	% Planting	CCI among planters	% selling among planters	CCI conditional on planting and selling
*Malawi*				

Maize	97.4	5.0	84.2	5.9
Cassava	11.0	4.3	60.8	7.1
Ground Nut	27.1	29.1	88.1	33.1
Tobacco	14.8	90.5	95.1	95.2
Soya	5.6	43.0	76.8	56.0
Pigeon Peas	22.1	15.1	58.3	26.0
Beans	11.1	10.1	37.8	26.8
Food crops	99.7	9.9	88.1	11.3
Non-food crops	16.8	89.8	94.2	95.3

*Tanzania*				

Maize	78.3	15.6	53.8	29.0
Ground Nut	14.4	28.3	42.6	66.5
Paddy Rice	19.8	30.7	56.0	54.8
Beans	28.7	19.9	35.0	57.0
Sorghum	11.1	12.7	24.2	52.4
Sweet Potato	9.9	11.2	20.8	53.9
Cowpeas	6.8	19.4	26.9	72.0
Food crops	99.2	23.8	64.8	36.7
Non-food crops	9.3	85.9	88.6	97.0

*Uganda*				

Maize	58.4	20.0	54.7	36.5
Cassava	41.4	8.0	19.8	40.3
Ground Nut	26.3	21.2	61.5	34.5
Banana (food)	49.6	34.4	67.3	51.2
Sweet Potato	42.8	5.5	13.9	39.9
Coffee	18.8	86.7	87.6	98.9
Beans	65.0	13.2	33.6	39.3
Food crops	99.7	22.5	76.3	29.5
Non-food crops	21.2	92.1	94.6	97.4

**Table 5:** CCI quintile breakdown of nutritional outcomes.

			Nutritional measure							
			HAZ	WAZ	WHZ	Stunted	Wasted	Underweight	Food Expenditure ($)	Kilo Calories
Malawi	CCI Quintile	No Sales	−1.31	−0.52	0.29	25.6	3.9	5.7	0.42	2418
		1	−1.22	−0.48	0.28	25.2	3.0	4.7	0.47	2352
		2	−1.53	−0.57	0.41	32.8	2.7	7.5	0.53	2546
		3	−1.32	−0.41	0.46	30.3	3.6	5.5	0.54	2670
		4	−1.40	−0.54	0.35	30.5	4.7	6.8	0.55	2538
		5	−1.52	−0.51	0.47	36.5	3.8	7.7	0.57	2640
	Country mean		−1.39	−0.57	0.39	30.7	3.6	6.4	0.52	2536

Tanzania	CCI Quintile	No Sales	−1.72	−0.95	0.02	42.6	5.7	14.4	0.63	1972
		1	−1.81	−1.10	−0.15	43.4	7.7	24.1	0.66	2215
		2	−1.85	−0.97	0.10	47.2	7.1	16.9	0.59	2051
		3	−1.67	−1.02	−0.13	45.1	6.2	15.5	0.61	2004
		4	−1.62	−0.88	0.03	40.1	5.5	12.1	0.62	2074
		5	−1.58	−0.92	−0.06	32.4	5.5	14.0	0.64	2044
	Country mean		−1.71	−0.96	−0.02	41.9	6.2	15.6	0.63	2044

Uganda	CCI Quintile	No Sales	−1.43	−0.83	−0.04	32.1	2.4	14.5	0.35	1954
		1	−1.35	−0.58	0.26	36.8	1.7	9.4	0.40	2229
		2	−1.85	−1.01	0.07	45.6	6.1	16.1	0.41	2299
		3	−1.59	−0.78	0.18	37.0	5.1	12.3	0.44	2546
		4	−1.57	−0.70	0.27	31.8	1.6	10.7	0.40	2362
		5	−1.44	−0.58	0.31	34.4	2.4	7.9	0.44	2132
	Country mean		−1.53	−0.75	0.16	36.0	3.2	11.9	0.40	2243

Note: Food expenditure and kilo calorie data are per capita, and at the household level.

**Table 6:** Individual fixed effects specification for pooled sample.

HAZ	(1)	(2)	(3)	(4)	(5)
ln(pc. Expenditure)	0.108	0.115	0.110	0.115	0.107
	(0.0737)	(0.0742)	(0.0742)	(0.0735)	(0.0737)
CCI	0.178	0.160		0.155	
	(0.123)	(0.121)		(0.122)	
Perc. Female		0.00186			
		(0.00118)			
Female CCI			0.298		
			(0.213)		
Perc. Food				0.00108	
				(0.000895)	
Food CCI					0.201
					(0.134)
Adjusted R2	0.097	0.099	0.097	0.099	0.097

WAZ	(1)	(2)	(3)	(4)	(5)

ln(pc. Expenditure)	0.0579	0.0502	0.0565	0.0639	0.0577
	(0.0518)	(0.0503)	(0.0520)	(0.0519)	(0.0518)
CCI	0.0620	0.0816		0.0409	
	(0.0871)	(0.0872)		(0.0863)	
Perc. Female		−0.00195^∗∗^			
		(0.000864)			
Female CCI			0.00839		
			(0.138)		
Perc. Food				0.000950^∗^	
				(0.000533)	
Food CCI					0.0827
					(0.0954)
Adjusted R2	0.097	0.099	0.097	0.099	0.097

WHZ	(1)	(2)	(3)	(4)	(5)

ln(pc. Expenditure)	0.0148	−0.000592	0.0118	0.0187	0.0152
	(0.0828)	(0.0809)	(0.0835)	(0.0825)	(0.0826)
CCI	−0.0462	−0.00688		−0.0600	
	(0.133)	(0.130)		(0.132)	
Perc. Female		−0.00391^∗∗^			
		(0.00151)			
Female CCI			−0.199		
			(0.265)		
Perc. Food				0.000622	
				(0.000787)	
Food CCI					−0.0396
					(0.155)
Adjusted R2	0.031	0.039	0.032	0.032	0.031

Observations	3140	3140	3140	3140	3140

^⁎⁎⁎^ *p* < 0.01; ^⁎⁎^ *p* < 0.05; ^⁎^ *p* < 0.1.

**Table 7:** Random effects logit specification for pooled sample.

Stunted	(1)	(2)	(3)	(4)	(5)
ln(pc. Expenditure)	−0.242^∗∗^	−0.242^∗∗^	−0.243^∗∗^	−0.247^∗∗^	−0.244^∗∗^
	(0.109)	(0.109)	(0.109)	(0.110)	(0.109)
CCI	0.179	0.188		0.191	
	(0.239)	(0.240)		(0.239)	
Perc. Female		−0.000817			
		(0.00228)			
Female CCI			0.165		
			(0.441)		
Perc. Food				−0.00138	
				(0.00136)	
Food CCI					−0.0405
					(0.263)

Wasted	(1)	(2)	(3)	(4)	(5)

ln(pc. Expenditure)	−0.176	−0.185	−0.180	−0.183	−0.183
	(0.189)	(0.191)	(0.190)	(0.191)	(0.189)
CCI	0.161	0.284		0.171	
	(0.436)	(0.434)		(0.429)	
Perc. Female		−0.0112^∗∗^			
		(0.00487)			
Female CCI			−0.688		
			(1.097)		
Perc. Food				−0.00279	
				(0.00221)	
Food CCI					0.526
					(0.448)

Underweight	(1)	(2)	(3)	(4)	(5)

ln(pc. Expenditure)	−0.692^∗∗∗^	−0.692^∗∗∗^	−0.698^∗∗∗^	−0.700^∗∗∗^	−0.695^∗∗∗^
	(0.168)	(0.168)	(0.168)	(0.169)	(0.167)
CCI	0.315	0.326		0.334	
	(0.362)	(0.365)		(0.361)	
Perc. Female		−0.000952			
		(0.00402)			
Female CCI			−0.238		
			(0.748)		
Perc. Food				−0.00234	
				(0.00212)	
Food CCI					0.386
					(0.395)

Observations	3140	3140	3140	3140	3140

^⁎⁎⁎^ *p* < 0.01; ^⁎⁎^ *p* < 0.05; ^⁎^ *p* < 0.1.

**Table 8:** Household fixed effects specification for nat. log of household’s per capita food expenditure.

Malawi	(1)	(2)	(3)	(4)	(5)
ln(harvest value)	0.103^∗∗∗^	0.102^∗∗∗^	0.102^∗∗∗^	0.102^∗∗∗^	0.102^∗∗∗^
	(0.0186)	(0.0185)	(0.0175)	(0.0185)	(0.0176)
CCI	0.0132	−0.00158		0.0172	
	(0.0842)	(0.0842)		(0.0824)	
Perc. Female		0.000628			
		(0.000522)			
Female CCI			0.202		
			(0.163)		
Perc. Food				0.000223	
				(0.000383)	
Food CCI					0.0791
					(0.0824)
Adjusted R2	0.347	0.348	0.348	0.347	0.347
Observations	4444	4444	4444	4444	4444

Tanzania	(1)	(2)	(3)	(4)	(5)

ln(harvest value)	0.0436^∗∗∗^	0.0439^∗∗∗^	0.0396^∗∗∗^	0.0469^∗∗∗^	0.0437^∗∗∗^
	(0.0121)	(0.0121)	(0.0115)	(0.0131)	(0.0119)
CCI	−0.0733	−0.0657		−0.0552	
	(0.0588)	(0.0601)		(0.0588)	
Perc. Female		−0.000311			
		(0.000599)			
Female CCI			−0.105		
			(0.104)		
Perc. Food				−0.000399	
				(0.000366)	
Food CCI					−0.0856
					(0.0611)
Adjusted R2	0.130	0.130	0.130	0.131	0.130
Observations	3488	3488	3488	3488	3488

Uganda	(1)	(2)	(3)	(4)	(5)

ln(harvest value)	0.0529^∗^	0.0527	0.0506	0.0498	0.0530
	(0.0319)	(0.0322)	(0.0309)	(0.0325)	(0.0321)
CCI	−0.132^∗^	−0.133^∗^		−0.146^∗^	
	(0.0754)	(0.0744)		(0.0764)	
Perc. Female		4.21e−05			
		(0.000575)			
Female CCI			−0.202^∗∗^		
			(0.102)		
Perc. Food				0.000644	
				(0.000473)	
Food CCI					−0.133
					(0.0841)
Adjusted R2	0.134	0.134	0.136	0.136	0.134
Observations	3174	3174	3174	3174	3174

^⁎⁎⁎^ *p* < 0.01; ^⁎⁎^ *p* < 0.05; ^⁎^ *p* < 0.1.

**Table 9:** Household fixed effects specification for nat. log of household total per capita expenditure.

Malawi	(1)	(2)	(3)	(4)	(5)
ln(harvest value)	0.0977^∗∗∗^	0.0978^∗∗∗^	0.0997^∗∗∗^	0.0973^∗∗∗^	0.0990^∗∗∗^
	(0.0167)	(0.0166)	(0.0160)	(0.0166)	(0.0159)
CCI	0.0566	0.0605		0.0600	
	(0.0686)	(0.0687)		(0.0674)	
Perc. Female		−0.000163			
		(0.000425)			
Female CCI			0.0442		
			(0.137)		
Perc. Food				0.000185	
				(0.000306)	
Food CCI					0.120
					(0.0728)
Adjusted R2	0.444	0.445	0.444	0.445	0.445
Observations	4444	4444	4444	4444	4444

Tanzania	(1)	(2)	(3)	(4)	(5)

ln(harvest value)	0.0319^∗∗∗^	0.0330^∗∗∗^	0.0309^∗∗∗^	0.0357^∗∗∗^	0.0324^∗∗∗^
	(0.0105)	(0.0105)	(0.0100)	(0.0112)	(0.0103)
CCI	−0.0416	−0.0178		−0.0212	
	(0.0576)	(0.0585)		(0.0574)	
Perc. Female		−0.000969^∗^			
		(0.000507)			
Female CCI			−0.163		
			(0.0994)		
Perc. Food				−0.000450	
				(0.000321)	
Food CCI					−0.0555
					(0.0596)
Adjusted R2	0.130	0.132	0.131	0.131	0.130
Observations	3488	3488	3488	3488	3488

Uganda	(1)	(2)	(3)	(4)	(5)

ln(harvest value)	0.0164	0.0162	0.0154	0.0130	0.0146
	(0.0222)	(0.0223)	(0.0210)	(0.0225)	(0.0223)
CCI	−0.0539	−0.0552		−0.0704	
	(0.0805)	(0.0799)		(0.0809)	
Perc. Female		5.46e−05			
		(0.000463)			
Female CCI			−0.0826		
			(0.0942)		
Perc. Food				0.000726^∗^	
				(0.000421)	
Food CCI					−0.0156
					(0.0890)
Adjusted R2	0.225	0.225	0.226	0.228	0.225
Observations	3174	3174	3174	3174	3174

^⁎⁎⁎^ *p* < 0.01; ^⁎⁎^ *p* < 0.05; ^⁎^ *p* < 0.1.

## Appendix A

### A.1 Adjustment of crop production sold

Obtaining figures for household commercialization is one of the aims of this study. Households in our samples were interviewed at different points of the year, thus households in the sample differ in the time span during which they could have sold their harvest. Additionally, different crops have different harvest periods throughout the season. These two facts complicate assessing whether or not a household sells crop, and if so, how much would it sell if it had sufficient time. To proceed, we utilize an adjustment procedure similar to that detailed in Kaminski and Christiaensen (2014), which was used to estimate post-harvest loss.

The analysis is conducted at the crop level. The sample is additionally separated into crop groupings, depending on the country and crop type.^36^ Table A1 gives a breakdown of the crops considered by country. Vegetables and fruits are not considered due to their short shelf life. Also not considered are goods that are inherently commercial in nature and the household has produced for the market: these are all non-food items. Examples of non-food items include cotton, tobacco and coffee.

For each crop, the sample is initially analyzed only for those who reported sales of the crop by the time of interview. For these observations, the goal is to determine how much they would have sold given enough time, or if they would have sold more given more time. The following regression is estimated for each crop:

(1) y=Xβ+f(t)+δ(Hht)+ε

In this specification, y is T×1 vector of the natural logarithm of the kilos sold by the household, β is a K×1 vector of parameters to be estimated including a constant term, and X is a T×K matrix. Among the considered regressors are a set of household characteristics, including characteristics for the head of the household. Time span between harvest and interview is considered in Hht(harvestedkilospercapita×timespan). Additionally, time is also considered in f(t) which is a T×1 vector and is equal to:

(2) $$f(t)=\theta t+\gamma t^{2}$$

Finally, ε is the random error term and is assumed to be normal, independent and identically distributed (i.i.d.) with mean zero.

In order to get the adjusted sales value, the time span which maximizes kilos sold must be obtained. For this to be the case, it is necessary that the second derivative of the estimated model be negative. Therefore, the adjustment is done only for the crops where this holds true. The final sales value is the original sales value for all households for which the time span is greater than the optimal t. For households where the time span is less than the optimal t, the adjusted sales value is equal to the original sales value and the difference between the expected sales valued at the optimal time and the expected sales value at the actual time.

The other portion of the analysis consists in determining if and how much households that did not report sales at the time of interview would have sold. The probability of selling crops is estimated using a probit model given the reported time span, and utilizes the same set of regressors as in Eq. (1). Given the estimated parameters, once again it is necessary to find the time span that maximizes the probability of selling the given crop. Therefore, it is necessary that the second derivative of the function with respect to time span be negative. If this holds, then the adjustment for that crop is done for households for which the time span is less than or equal to the estimated optimal time span. For these households, the predicted probability of selling crops is equal to:

(3) $$P({\mathrm{sell}}_{i}=1)=P({\mathrm{sell}}_{i}=1|X_{i}\text{,}t^{*})-P({\mathrm{sell}}_{i}=1|X_{i}\text{,}t=t_{i})$$

Using this probability, it is possible to obtain a predicted sales value for these households which is equal to:

(4) $$\left[E(y_{i}|X_{i}\text{,}t^{*}\text{,}{\mathrm{sell}}_{i}=0)-E(y_{i}|X_{i}\text{,}t_{i}\text{,}{\mathrm{sell}}_{i}=0)]\times P({\mathrm{sell}}_{i}=1\right)$$

Once these values are obtained it is possible to obtain the adjusted CCI for each household. The results from the estimations are available from the authors upon request.

## Footnotes

1 See for instance Hernandez et al., 1974, Lappe, 1977, as well as Dewey (1981).

2 For a quick overview of the several areas of debate of agricultural commercialization over time, see for example Maxwell and Fernando (1989) or the more recent Wiggins et al. (2011).

3 Wiggins et al. (2011) mention that this may be somewhat overblown, since in most cases small farmers tend to prioritize growing their main staple food.

4 Agricultural commercialization was often presented as the result of colonialist-type rural policies, favoring “cash crops” mainly for export reasons with minimal advantages for the rural population. This line of argument was favored by researchers supporting the so-called “food-first” view. For a more detailed review of this line of argument against cash cropping and related sources, see Maxwell and Fernando (1989), Appendix A.

5 Other relevant research projects were also carried out in this period by the Department of Agricultural Economics of the Michigan State University. See for instance, Lev (1981).

6 Previous literature was limited in its scope by the available data, since information had been mainly available in aggregate form.

7 See in particular the empirical results provided in von Braun and Kennedy (1986).

8 The IFPRI research agenda on agricultural commercialization and nutrition stretched from the mid-1980s to the mid-1990s.

9 The study by Wood et al. (2013) is a notable exception. Others (Carletto et al., 1999, Carletto et al., 2010, Carletto et al., 2011) focused more on the determinants of the commercialization process and its impact on poverty, as opposed to food security and nutrition.

10 A wider literature review on studies conducted before the mid-1980s was carried out six years later by Randolph (1992). It showed similar results.

11 See in particular Pinstrup-Andersen (1983).

12 von Braun and Kennedy (1986).

13 In this paper, we use different anthropometric measures of children under five years of age to compute measures of stunting, wasting and underweight as well as associated Z-scores capturing deviations of sample children from a reference population.

14 Indeed, “where farmers are free to make their own production decisions, increased commercialization will occur only if the farmer does not perceive that another production option would more effectively achieve his goals within the constraints it faces. Thus, although higher income is only one of a set of possible goals, it is highly unlikely that a farmer would produce export crops unless he or she expects it to yield economic gains than any other realistic production option” (see von Braun and Kennedy, 1986).

15 Additionally it is possible that different household members have different income elasticities among food products.

16 Von Braun et al. (1989) also observe that in some cases malnutrition is endemic in a given country; so that households are not always aware of the problem (“their children look like most other children in the community”).

17 Von Braun et al. (1989) and von Braun and Kennedy (1994). The first round of country case studies, summarized in von Braun et al. (1989) included Guatemala, Kenya, Rwanda, the Philippines, and Gambia. In these countries, rural households had recently undergone a change from semi-subsistence food production to increased production of crops for sales, thereby representing an ideal study area of the impact of agricultural commercialization. These country studies were subsequently complemented with 5 more field studies in India, Malawi, Papua New Guinea, Sierra Leone and Zambia.

18 To be more precise, a positive impact was found in all countries except for Kenya, where the effect was deemed neutral. A positive impact was recorded also in four of the five studies carried out in the early 1990s, with the exception of Sierra Leone, where a deterioration of the nutritional status was detected. See Bellin (1994).

19 At the macroeconomic level trade policies, market reforms, improvement in rural infrastructure, as well as the development of legal and contractual environments are seen as crucial to promoting an inclusive commercialization process. At the microeconomic level, instead, two important policy areas are identified in the setting up of effective rural financial institutions and in the provision of extension services at the household level to help farmers to avoid crop management failures. Furthermore, the development and promotion of community health and sanitation services is highly recommended to maximize the health and nutrition returns of increased income. Finally, promotion of technological change in food crops is advocated in order to enhance food security at the household level.

20 For example when households adopt new crops and do not obtain the expected gains. This is detailed in von Braun and Kennedy (1994); an example is when there is inelastic demand, yet many producers enter the market and reduce crop prices.

21 See also Billah (2002) on the links between crop production and nutrition in Bangladesh.

22 See http://www.worldbank.org/lsms. Other countries covered under the program include Ethiopia, Mali, Niger, Nigeria.

23 Additional information and data from the imputation are available upon request.

24 In this study, livestock ownership has been excluded from the sample.

25 More detailed information on the surveys, including sample size and survey design can be found at www.worldbank.org/lsms.

26 See Appendix A.

27 Randolph (1992), p. 11.

28 In some case this choice was justified by the early stage of the process of agricultural commercialization in the country under review, with a negligible share of farmers resorting to purchased inputs. See for instance Randolph (1992).

29 Randolph (1992), p. 291.

30 In more detail, our dataset provides information as to who in the household decides what to do with the earnings from sales of a crop. This was used to determine male and female revenues from crop sales within the household. Surveys also provide information on who in the household manages a plot. This was used to determine the harvest value for each gender within the household. The CCI by gender was thus computed as the percentage of each gender’s harvest (in monetary terms) which was reported to have been sold.

31 Results by gender not reported and available upon request.

32 Figures lower than 100% for tobacco in Malawi are likely to reflect accumulation of stocks.

33 For example in Cote d’Ivoire, Duflo and Udry (2004) find that income shocks that benefitted females were positively related to household food expenditure.

34 Simultaneity is especially a strong concern, since we observe expenditures at a point when the household may not have completed its commercialization process yet.

35 We also run a specification where we include the household’s revenue from the sale of crops, instead of the CCI, and the coefficient is also not significant (results available upon request).
